# Effects of the Genetic Depletion of Polysialyltransferases on the Structure and Connectivity of Interneurons in the Adult Prefrontal Cortex

**DOI:** 10.3389/fnana.2019.00006

**Published:** 2019-02-06

**Authors:** Yasmina Curto, Julia Alcaide, Iris Röckle, Herbert Hildebrandt, Juan Nacher

**Affiliations:** ^1^Neurobiology Unit, Department of Cell Biology, Interdisciplinary Research Structure for Biotechnology and Biomedicine (BIOTECMED), Universitat de València, Valencia, Spain; ^2^Institute of Clinical Biochemistry, Hannover Medical School, Hannover, Germany; ^3^Centro de Investigación Biomédica en Red de Salud Mental (CIBERSAM): Spanish National Network for Research in Mental Health, Madrid, Spain; ^4^Fundación Investigación Hospital Clínico de Valencia, INCLIVA, Valencia, Spain

**Keywords:** polysialic acid (polysia), interneuron, basket cell, polysialyltransferases, prefrontal cortex, dendritic arborization

## Abstract

Polysialic acid (polySia) is a complex sugar that in the nervous system appears mainly as a posttranslational modification of the neural cell adhesion molecule (NCAM). PolySia plays important roles during brain development, but also in its plasticity during adulthood. Two polysialyltransferases (polyST), ST8SIA2 and ST8SIA4, are involved in the synthesis and attachment of polySia. Both polyST are relevant for developmental migration of cortical interneurons and their establishment in the prefrontal cortex (PFC). In contrast, only ST8SIA4 appears to be important for the structural plasticity of a subpopulation of cortical interneurons in the adult. Interestingly, *ST8SIA2* and NCAM are candidate genes for schizophrenia, a disorder in which interneuronal circuits are altered. However, there is still no data on the effects of polyST depletion on the dendritic structure or the connectivity of cortical interneurons. Here, we studied the contribution of each polyST on these parameters in the medial PFC (mPFC) of polyST knock-out mice with GAD67-GFP-labeled interneurons. Genetic depletion of ST8SIA4, but not ST8SIA2, resulted in a decrease in the complexity of the dendritic arbor of interneurons. In contrast, ablation of either of the two polyST induced a decrease in the density of parvalbumin (PV) expressing perisomatic puncta on pyramidal neurons. Thus, the depletion of each polyST results in similar impairments of not only developmental migration but also efferent synaptic connectivity of interneurons. In contrast, the loss of ST8SIA4 has a unique effect on dendritic structure, hence on afferent connectivity, suggesting differential and independent contributions of each polyST to neuritogenesis and synaptogenesis.

## Introduction

Polysialic acid (polySia) is a cell-surface glycan, which in the brain appears almost exclusively attached to the neural cell adhesion molecule (NCAM; Gómez-Climent et al., 2011; Mühlenhoff et al., 2013). The presence of polySia creates steric impediments, which modulate homo- and heterophilic cell-cell and cell-matrix interactions, leading to decreased adhesion (Rutishauser, 2008). Therefore, polySia is involved in several neurodevelopmental processes such as neuroblast migration, neurite outgrowth, axonal pathfinding, axon tract formation, as well as synaptogenesis (Rutishauser, 2008; Schnaar et al., 2014).

Two independently regulated polysialyltransferases (polyST), named ST8SIA2 and ST8SIA4, are in charge of the synthesis and attachment of polySia to NCAM. The expression of both polySTs shows a partial overlap but distinct time course during development (Hildebrandt et al., 2007). ST8SIA2 expression is prominent in embryonic and perinatal stages, but subsequently its levels decrease rapidly and remain low in young and adult animals. By contrast, the level of ST8SIA4 expression, although reduced as well, remains higher and more persistent after early postnatal development (Hildebrandt et al., 1998; Ong et al., 1998; Oltmann-Norden et al., 2008).

To investigate more accurately the contribution of each polyST, single ST8SIA2 and ST8SIA4 knock-out mice have been developed. The study of the cerebral cortex of adult mice from these strains revealed that ST8SIA4 is exclusively responsible for the addition of polySia to NCAM in a subpopulation of mature interneurons (Nacher et al., 2010). Interestingly, the cortical interneurons that express polySia have reduced structural features and connectivity, suggesting an insulating role for this molecule (Gómez-Climent et al., 2011; Nacher et al., 2013). PolySia is expressed by different subtypes of cortical interneurons, including parvalbumin (PV)-positive basket cells. In this interneuron subtype polySia is found in some of the synapses that these cells establish on the perisomatic region of pyramidal cells (Castillo-Gómez et al., 2008). In fact, the depletion of polySia *in vivo* and *in vitro* using the enzyme Endo-Neuraminidase-N (Endo-N) has shown that the expression of this complex sugar is of paramount importance in the regulation of this inhibitory input (Castillo-Gómez et al., 2011, 2016). Moreover, the postnatal decrease in polySia expression is critical for inhibitory circuit maturation and critical period plasticity in the visual cortex (Di Cristo et al., 2007). In the adult cerebral cortex polySia is also expressed in the subpopulation of interneurons expressing somatostatin, which target the distal dendrites of pyramidal neurons and are characterized by the presence of dendritic spines (Gómez-Climent et al., 2011). Interestingly, the depletion of polySia alters the density of these postsynaptic elements (Guirado et al., 2014a). Altogether, these previous results indicate an important role for polySia in regulating the morphology and connectivity of inhibitory neurons in the adult brain.

PolySia also has an important role in interneuronal development. The manipulation of polySia levels by the genetic depletion of either of the two polySTs affects the migratory capacity and the final density of cortical interneurons, including PV and somatostatin expressing cells (Kröcher et al., 2014). However, it is not known whether genetic depletion of polySTs has an impact on the neuritogenesis and synaptogenesis of these interneurons, which may lead to alterations in their structure or connectivity in the adult brain. This is particularly important because alterations in cortical inhibitory networks, especially those of the prefrontal cortex (PFC) appear to be involved in the etiopathology of certain mental disorders, particularly schizophrenia (Marín, 2012). Moreover, in human patients and in animal models, several studies have shown alterations in polySia expression and genetic associations of *NCAM1* and *ST8SIA2* variants with schizophrenia (Varea et al., 2007; Anney et al., 2010; Brennaman and Maness, 2010; Gilabert-Juan et al., 2011; McAuley et al., 2012; Guirado et al., 2014b; Castillo-Gómez et al., 2016, 2017).

Here, we asked whether polySTs, apart from migration, are also crucial for neuritogenesis and/or synaptogenesis of cortical interneurons. To this end, we analyzed the structure and connectivity of interneurons in the PFC, specifically in the prelimbic and infralimbic cortices, of adult ST8SIA2 and ST8SIA4 knock-out mice with GAD67-GFP-labeled interneurons. The dendritic structure was studied by Sholl analysis and synaptic connectivity was addressed by evaluations of inhibitory perisomatic puncta that PV expressing basket cells establish around the somata of pyramidal neurons.

## Materials and Methods

All animal experimentation was conducted in accordance with the Directive 2010/63/EU of the European Parliament and of the Council of 22 September 2010 on the protection of animals used for scientific purposes and was approved by the Committee on Bioethics of the Universitat de València. Every effort was made to minimize the number of animals used and their suffering. C57BL/6J and mutant mice were bred at the central animal facility at Hannover Medical School. *St8sia2* and *St8sia4* knockout strains, backcrossed with C57BL/6J mice for six generations, were cross-bred with GAD67-GFP knock-in mice (Tamamaki et al., 2003) to obtain *St8sia2*^−/−^ or *St8sia4*^−/−^ mice heterozygous for the transgene (*St8sia2*^−/−^ GAD67-GFP and *St8sia4*^−/−^ GAD67-GFP). The control group consisted of Gad67-GFP positive St8sia2^+/+^ and St8sia4^+/+^ mice derived from the same founder colonies as the knockout animals. Genotyping was performed by PCR as previously described (Tamamaki et al., 2003; Weinhold et al., 2005). Six animals per group were used to perform the different experiments.

All mice were perfused transcardially when 3 months old, first for 1 min with NaCl 0.9% and then for 30 min with 4% paraformaldehyde in sodium phosphate buffer 0.1 M, pH 7.4. To analyze the dendritic arborization of GFP-expressing, the right hemispheres were sectioned in coronal 100-μm thick sections using a vibratome (Leica VT 1000E) and collected in three subseries. For the immunohistochemical assays, the left hemispheres were frozen and cryoprotected in 30% sucrose in PB 0.1 M. Then, coronal sections (50 μm) were obtained with a freezing-sliding microtome (Leica SM2000R) and collected in six subseries.

In order to characterize neurochemically the GAD67-GFP interneurons in the mPFC, we performed double immunostainings using free-floating 50 μm sections tissue of thickness from the different strains of mice: we used anti-GFP primary antibody in combination with anti-: (a) anti-PV; (b) calretinin (anti-CR); and (c) calbindin (anti-CB) primary antibodies (see Table 1). Briefly, sections were washed in PBS and then incubated for 1 h in 10% normal donkey serum (NDS; Jackson ImmunoResearch Laboratories) in PBS with 0.2% Triton X-100 (PBST; Sigma-Aldrich). Afterwards, sections were incubated for 48 h at 4°C with the primary antibodies diluted in PBST and 5% NDS (Table 1). After washing, sections were incubated for 2 h at room temperature with the appropriate secondary antibodies diluted in PBST and 5% NDS (Table 1). Finally, sections were washed in PB 0.1 M, mounted on slides, and coverslipped using fluorescence mounting medium (Dako Diagnósticos).

**Table 1:** Primary and secondary antibodies used in the study.

Anti	Host	Isotype/Label	Dilution	Incubation	Company
**Primary antibodies**					
GFP	Chicken	Ig Y	1:1,000	48 h, 4°C	Abcam
PV	Guinea Pig	Ig G	1:2,000	48 h, 4°C	Synaptic Systems
CR	Rabbit	Ig G	1:2,000	48 h, 4°C	Swant
CB	Mouse	Ig G1	1:5,000	48 h, 4°C	Sigma Life Science
CaMKII-α	Mouse	Ig G1	1:500	48 h, 4°C	Abcam
SYN	Rabbit	Ig G	1:1,000	48 h, 4°C	Chemicon-Millipore
**Secondary antibodies**					
Chicken IgY	Donkey	CF 488	1:400	2 h, 25°C	Sigma Life Science
Guinea pig IgG	Goat	Alexa 555	1:400	2 h, 25°C	Life Technologies
Rabbit IgG	Donkey	Alexa 555	1:400	2 h, 25°C	Life Technologies
Mouse IgG1	Goat	Alexa 635	1:400	2 h, 25°C	Life Technologies
Mouse IgG1	Goat	Dylight 649	1:400	2 h, 25°C	Jackson ImmunoResearch
Guinea pig IgG	Donkey	Biotin	1:400	2 h, 25°C	Jackson ImmunoResearch
Streptavidin		Dylight 405	1:400	1 h, 25°C	Life Technologies

For the structural analysis, a simple fluorescent immunohistochemistry against GFP was performed using coronal sections of 100 μm microns. The immunohistochemical protocol was similar to that described above, using a polyclonal chicken IgY anti-GFP, antibody to amplify the GFP fluorescent signal in interneurons destined to morphological studies (Table 1).

In order to analyze the density of perisomatic puncta surrounding pyramidal neurons, after washing, coronal 50 μm-thick sections were first treated for 1 min with an antigen unmasking solution (0.01 M citrate buffer, pH 6) at 100°C. After cooling down sections to room temperature, they were processed for immunofluorescence as described above with a cocktail of primary antibodies (see Table 1): including anti-CaMKII-α, anti-synaptophysin (SYN) and anti-PV (1:2,000, Synaptic Systems). After being washed, sections were incubated with the appropriate fluorescent secondary antibodies (see Table 1). Sections incubated with Donkey anti-Guinea pig-biotinylated antibodies were subsequently incubated for 1 h in Streptavidin (Life Technologies). Finally, sections were mounted on slides and coverslipped using fluorescence mounting medium (Dako Diagnósticos).

All studied sections passed through all procedures simultaneously in order to minimize any difference from the immunohistochemical staining itself. To avoid any bias in the analysis, all slides were coded prior to analysis and remained coded until the experiment was completed.

Sections double-labeled for GFP and interneuronal subpopulation markers (PV, CB, CR) were observed under a confocal microscope (Leica TCS-SPE) using a 40× objective. Z-series of optical sections (0.2 μm apart) were obtained using sequential scanning mode and stacks were then processed using FIJI (ImageJ, NIH). Fifty GAD67-GFP-expressing neurons within the infralimbic and 50 within the prelimbic cortex were randomly selected from each animal and for each immunostaining to determine the co-expression of GAD67-GFP and each marker. Percentages of co-localization were determined for each animal and mean ± SEM were calculated.

Dendritic arborization was studied using confocal microscopy (Leica TCS-SPE) as previously described (Gilabert-Juan et al., 2011; Gómez-Climent et al., 2011). Z-series of optical sections (0.2 μm apart) covering the whole dendritic tree of selected interneurons were obtained using the sequential scanning mode and a 63× oil objective. Six animals of each genotype were used to perform the analysis. From each animal, six GAD67-GFP expressing neurons were selected from the prelimbic cortex and six from the infralimbic cortex with the soma located in layers II-III. In order to be analyzed, GFP-expressing cells had to fulfill the following features: (1) the cell must not show any truncated dendrites; (2) the dendritic arbor of the cell must show at least a process longer than 150 μm; and (3) the soma must be located at least 30 μm deep from the surface of the tissue. The stacks obtained were then processed using FIJI/ImageJ Software (Schindelin et al., 2012) to obtain 3D reconstructions (see Figure 2B). Neurons with highly overlapping dendritic tress were excluded from the analyses. The “Simple neurite tracer” tool was used to trace the dendrites of interest and allowed avoiding dendrites from surrounding neurons. Axons were also excluded by their more reduced thickness. The degree of dendritic arborization was analyzed using a procedure for deriving the Sholl profile (Gutierrez and Davies, 2007). The Sholl analysis consists on the measure of the number of intersections of the dendrites with spheres of increasing radius centered in the soma (Sholl, 1953). For each experimental group, mean ± SEM was determined and the resulting values were analyzed by one-way analyses of variance (ANOVA), with the number of neurons as the “*n*.” Previous Kolmogorov-Smirnov and Levene tests were performed to analyze the normality and homogeneity of variances, respectively. Significant differences were further analyzed by Bonferroni *post hoc* test, using the IBM SPSS statistics software (version 19).

**Figure 1:**
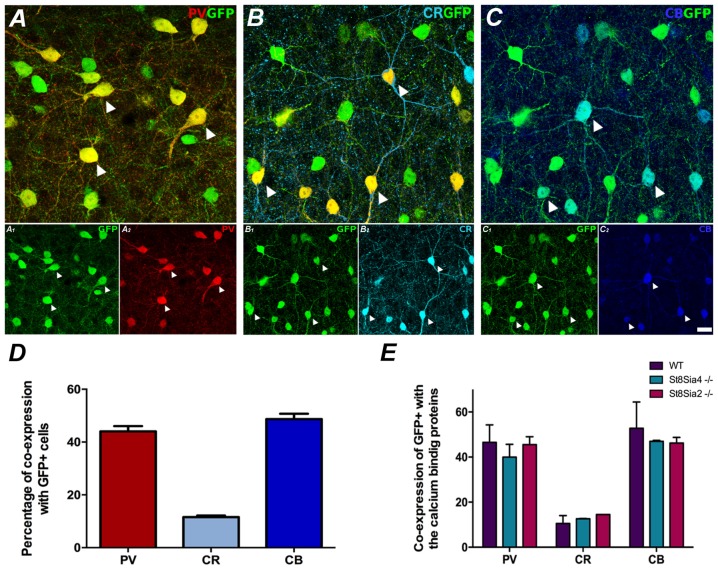
Neurochemical characterization of GAD67-GFP expressing neurons in the medial prefrontal cortex (mPFC); co-expression with the different calcium binding proteins: **(A)** Single confocal plane showing the expression of GAD67-GFP with parvalbumin (PV), **(B)** calretinin (CR) and **(C)** calbindin (CB). Arrowheads point to cells co-expressing GAD67-GFP and the different calcium binding proteins. **(D)** Graph showing the percentages of GAD67-GFP positive cells expressing these interneuronal markers. **(E)** Graph showing the similarity of these percentages of co-localization in the different strains of mice analyzed. Scale bar = 15 μm.

**Figure 2:**
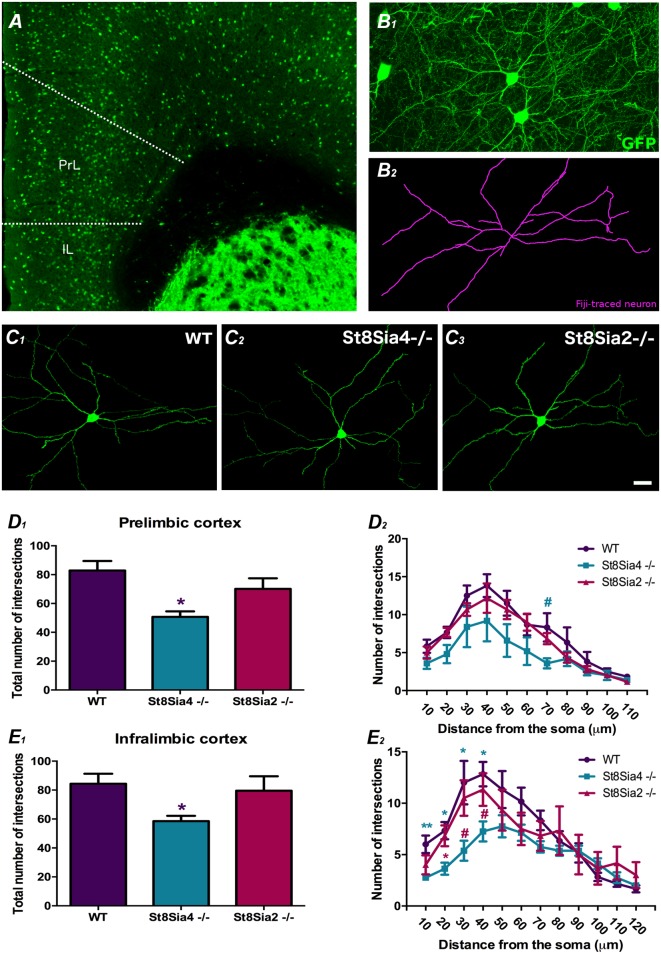
Analysis of dendritic arborization complexity in GAD67-GFP expressing interneurons in the mPFC. **(A)** Panoramic view of the prelimbic and infralimbic cortices showing the distribution of the GAD67-GFP labeled interneurons. **(B)** Higher magnification view of a GAD67-GFP expressing interneuron in green **(B_1_)** traced with FIJI software in purple **(B_2_)** for the study dendritic arborization (automated Sholl analysis). **(C_1–3_)** Detailed view of 3D reconstructions of GAD67-GFP expressing interneurons from wildtype, *St8Sia4*^−/−^ and *St8Sia2*^−/−^ mice. **(D_1_,E_1_)** Graphs showing statistically significant decreases in the total number of intersections in the *St8Sia4*^−/−^ strain compared with wildtypes in the prelimbic and infralimbic cortices. **(D_2_,E_2_)** Graph indicating that in the infralimbic cortex, the *St8Sia4*^−/−^ group also exhibits a significant decrease in the number of dendrite intersections analyzed with concentric spheres of 10 μm compared with both wildtypes and *St8Sia2*^−/−^ mice. No significant differences can be found in this parameter in the prelimbic cortex. *N* = 6 animals per group. From each animal, six GAD67-GFP expressing neurons were selected from the prelimbic cortex and 6 from the infralimbic cortex [one-way analyses of variance (ANOVA); ^#^0.1 > *p* > 0.05 for non-significant trends, *p < 0.05, **p < 0.01 for statistically significant values]. Scale bar = 100 μm for **(A)** and 15 μm for **(B,C)**.

The density of perisomatic puncta on pyramidal neurons was analyzed in the layers III and V of the prelimbic and infralimbic cortices using a confocal microscope (Olympus Fluoview FV 10i) and a 60× oil objective. Six animals per group were used for this study and 12 neurons in each of the prefrontocortical regions were counted per animal. The analyses were performed in sections corresponding to Bregma 3.08 mm/Interneural 6.88 mm and to Bregma −0.22 mm/Interneural 3.58 mm according to a mouse brain atlas (Paxinos and Franklin, 2008).

Confocal z-stacks covering the whole depth of the sections were taken with 1 μm step size and only subsets of confocal planes with the optimal penetration level for each antibody were selected. Images were processed using ImageJ software as follows (Guirado et al., 2018): the background was subtracted with rolling value of 50, converted to 8-bit deep images and binarized using a determined threshold value. This value depended on the marker and the area analyzed and was kept the same for all images with the same marker and area. Then, the images were processed with a blur filter to reduce noise and separate closely apposed puncta. Finally, values of puncta density for PV and SYN were obtained from each CaMKII-α expressing pyramidal neuron soma analyzed and expressed as number of puncta per micron of soma perimeter. For each experimental group, mean ± SEM was determined and the resulting values were analyzed by one-way ANOVA, with the number of neurons as the “*n*.” Previous Kolmogorov-Smirnov and Levene tests were performed to analyze the normality and homogeneity of variances, respectively. Significant differences were further analyzed by Tukey HSD or Games-Howell *post hoc* test depending on the results of the homogeneity test for variances.

## Results

### Characterization of GAD67-GFP Expressing Neurons in the mPFC

In order to understand in which subpopulation of interneurons we were performing the morphometric analyses, we analyzed the neurochemical phenotype of GFP-expressing interneurons in the prelimbic and infralimbic cortices. In all transgenic and wildtype mice used in this study GFP expressing neurons were mostly located in layers II, III and upper V, similar to what has been found in a previous study of the frontal motor cortex of GAD67-GFP knock-in mice (Tamamaki et al., 2003). Most of these neurons had multipolar or bipolar morphology. After careful observation of these GFP expressing interneurons in the mPFC of all the mice strains, we have found that none of them displayed degenerative symptoms such as swollen dendrites or axons, or the presence of abnormal nuclei. Interestingly, we could not observe dendritic spines in any of the dendritic arbors of the interneurons analyzed.

The GAD67-GFP expressing neurons in the prelimbic and infralimbic cortices mainly co-expressed PV and calbindin (CB; 44% ± 3.5 and 48.67% ± 3.56, respectively) and at a minor level CR (11.55% ± 1.08; Figures 1A–D). All the strains of mice analyzed had similar percentages of GAD67-GFP neurons co-expressing the different markers (Figure 1E; PV: *F*_(2,3)_ = 0.700, *p* = 0.563; CR: *F*_(2,3)_ = 1.170, *p* = 0.424; CB: *F*_(2,3)_ = 0.533, *p* = 0.634).

### Dendritic Arborization in GAD67-GFP Expressing Interneurons in the mPFC

The analysis of dendritic arbor complexity of GAD67-GFP expressing interneurons (Figures 2A,B_1–2_,C_1–3_) revealed a significant decrease in the total number of dendrite intersections in the prelimbic and infralimbic cortex of *St8Sia4*^−/−^ mice when compared with their wildtype littermates (Figures 2D_1_,E_1_; *F*_(2,14)_ = 3.941, *p* = 0.044 and *F*_(2,17)_ = 4.312, *p* = 0.040, respectively). Evaluation of the individual Sholl spheres indicated for the prelimbic cortex a non-significant trend towards a decrease in the 70 μm-radius (Figure 2D_2_; *F*_(2,17)_ = 3.344, *p* = 0.069) and for the infralimbic cortex, significant differences in the first 40 microns (Figure 2E_2_; 10 μm-radius, *F*_(2,17)_ = 5.991, *p* = 0.009; 20 μm-radius, *F*_(2,17)_ = 6.862, *p* = 0.012; 30 μm-radius, *F*_(2,17)_ = 5.284, *p* = 0.022; 40 μm-radius, *F*_(2,17)_ = 5.886, *p* = 0.014). Compared to *St8Sia2*^−/−^, *St8Sia4*^−/−^ mice displayed significantly less intersections in the 20 μm-radius of interneurons in the infralimbic cortex (*F*_(2,17)_ = 6.862, *p* = 0.031) and a non-significant trend towards a decrease in the 30 and 40 radii (*F*_(2,17)_ = 5.284, *p* = 0.093 and *F*_(2,17)_ = 5.886, *p* = 0.088, respectively). There were no differences between *St8Sia2*^−/−^ and wildtype mice.

### Density of Perisomatic Puncta on Medial Prefrontal Cortex Pyramidal Neurons

**Figure 3:**
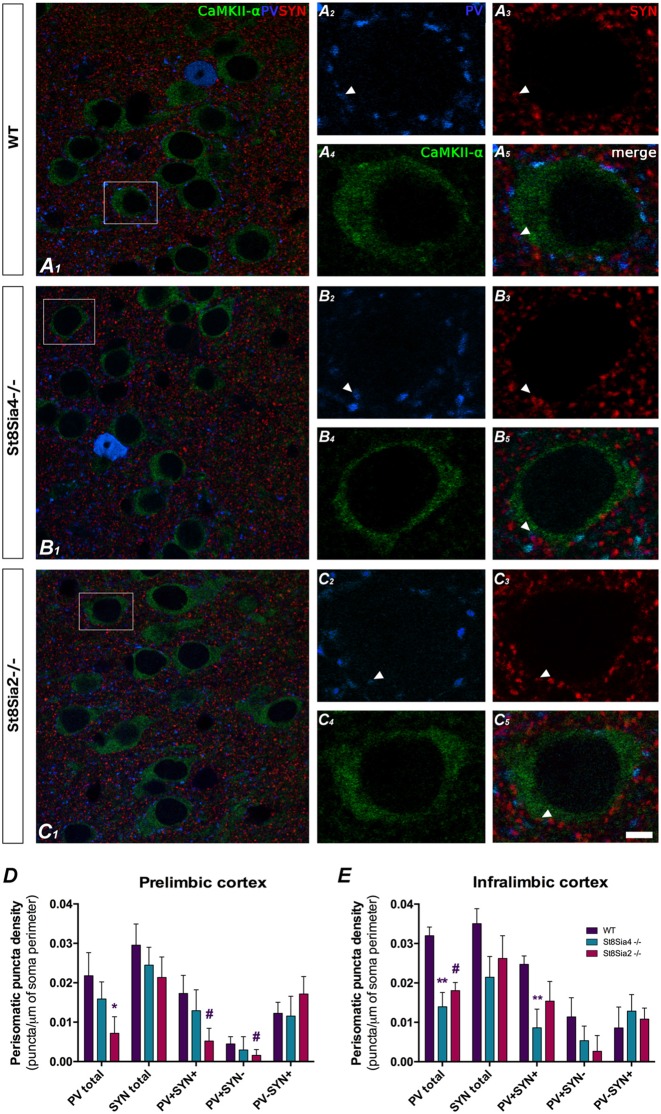
Confocal microscopic analysis of PV and synaptophysin (SYN) immunoreactive puncta surrounding CaMKII-α excitatory cell somata in the prelimbic and infralimbic cortices. **(A_1–5_, B_1–5_, C_1–5_)** Single confocal planes of pyramidal neurons somata (immunolabeled for CaMKII-α, green) showing changes in the perisomatic density of PV (blue) and SYN (red) immunoreactive puncta in the different strains of mice analyzed. Arrowheads indicate the co-localization between PV and SYN. **(D)** In the prelimbic cortex, graph shows a significant decrease in the density of PV expressing puncta in the *St8Sia2*^−/−^ mice when compared with wildtypes. **(E)** In the infralimbic cortex, the graph shows that *St8Sia4*^−/−^ mice have a decrease in the density of puncta expressing PV and of that co-expressing with PV and SYN. *N* = 6 animals per group (one-way ANOVA; ^#^0.1 > *p* > 0.05 for non-significant trends, *p < 0.05, **p < 0.01 for statistically significant values). Scale bar = 10 μm for **(A_1_,B_1_,C_1_)** and 5 μm for **(A_2–5_,B_2–5_,C_2–5_)**.

The study of the PV+ puncta on the perisomatic region of prefrontocortical pyramidal neurons revealed significant decreases in both knockout strains when compared to wildtype mice (Figures 3A_1–5_,B_1–5_,C_1–5_). In the prelimbic cortex of *St8Sia2*^−/−^ mice, the density of PV+ puncta was significantly lower (Figure 3D; *F*_(2,15)_ = 2.309, *p* = 0.033) and that of puncta co-expressing PV and SYN showed a non-significant trend towards a decrease (*F*_(2,15)_ = 1.722, *p* = 0.061). There was also a non-significant trend towards a decrease in the density of PV+/SYN− (*F*_(2,15)_ = 4.177, *p* = 0.086). In the infralimbic cortex of *St8Sia4*^−/−^ mice we observed significantly lower densities of PV+ (Figure 3E; *F*_(2,16)_ = 9.634, *p* = 0.004) and PV+/SYN+ (*F*_(2,15)_ = 6.369, *p* = 0.007) puncta. Additionally, in this region *St8Sia2*^−/−^ mice showed a non-significant trend towards a decrease in PV+ puncta (*F*_(2,16)_ = 9.634, *p* = 0.088).

## Discussion

This study demonstrates that the genetic depletion of ST8SIA4 affects the dendritic structure of prefrontocortical interneurons, whereas loss of either ST8SIA4 or ST8SIA4 causes reductions in the density of the perisomatic inhibitory puncta that a fraction of these cells establishes on pyramidal neurons. These data add to previous experiments demonstrating the impact of these enzymes on developmental migration and final densities of interneurons in the PFC (Kröcher et al., 2014).

The phenotype of the GFP+ interneurons analyzed in the mPFC in the present study is consistent with previous data for the motor cortex of GAD67-GFP mice (Tamamaki et al., 2003). Although we have not performed a detailed analysis of the different layers over the whole depth of the cortex, the percentages of GAD67-GFP positive cells expressing PV or CR are similar between the two studies. Unfortunately, we cannot establish a comparison for CB+ cells because this marker was not used in the other study.

An interesting finding of our study is that the percentages of the different subpopulations of interneurons, based on the expression of calcium binding proteins, were not altered by polyST depletion, although a previous study found a marked reduction in the density of PV+ and GFP+ cells in the mPFC of adult *St8Sia2*^−/−^ *and St8Sia4*^−/−^ mice (Kröcher et al., 2014). This simultaneous reduction of GFP+ and PV+ cells suggests a general reduction of interneuron densities in both polyST knockouts and explains why we did not detect different percentages of the PV+ subpopulation of GFP+ interneurons in wildtype controls and *St8Sia2*^−/−^ or *St8Sia4*^−/−^ mice.

In the present study we demonstrate that only the *St8Sia4*^−/−^ and not the *St8Sia2*^−/−^mice show a significant reduction in the dendritic arborization of the prefrontocortical interneurons. The 100 μm sections are thick enough to obtain a reasonable number of neurons that fulfill our requirements for inclusion in our analyses. However, the use of thicker sections would allow the quantification of wider dendritic arborizations and thus increase the precision of the analyses. This result from Sholl analysis suggests that the addition of polySia by ST8SIA4 is necessary for the correct development of the dendritic arbor of prefrontocortical interneurons. Further analyses need to be done to establish whether the reduction in dendritic arborization affects the excitatory or the inhibitory input that these cells receive or whether it has a similar impact on both types of afferences. Unfortunately, we were unable to differentiate between different subtypes of interneurons in our structural analysis. However, it has to be noted that we have not observed dendritic spines in the GFP+ interneurons analyzed, excluding that these were Martinotti cells (Gilabert-Juan et al., 2013b).

Considering that ST8SIA4 becomes predominant during postnatal maturation and is the only polyST to produce polySia in the adult cortex (Oltmann-Norden et al., 2008; Nacher et al., 2010), it is possible that the reduced dendritic arborization observed in prefrontocortical interneurons of *St8Sia4*^−/−^ mice is caused by the lack of this enzyme during neuritogenesis. Interestingly, similar effects on dendritic structure have been found in motor neurons of polySia-deficient *NCAM*^−/−^ mice, which exhibit a reduction in their dendritic field (Franz et al., 2005). Conversely, the enhancement of polySia expression increases neurite outgrowth in motoneurons derived from mouse embryonic stem cells (ESCs; El Maarouf et al., 2015). Future experiments should explore this putative effect of polySia on neuritogenesis by studying the structure of the dendritic arbor of interneurons during early postnatal development. Another, non-excluding, possibility is that the structural alterations observed were due to the absence of polySia in cortical interneurons after postnatal development. In fact, *St8Sia4* is the sole polyST responsible for the presence of polySia in adult cortical interneurons (Nacher et al., 2010). However, this possibility seems unlikely, because polySia is only present in the somata and dendrites of a small subpopulation of cortical interneurons (around 8% in the PFC). Moreover, a previous study from our laboratory showed that in the adult cerebral cortex polySia expressing interneurons have a reduced dendritic arborization when compared with interneurons lacking this molecule (Gómez-Climent et al., 2011). Nevertheless, it has to be noted that this study focused only on a subfraction of interneurons: those expressing somatostatin, which, as we have discussed above, are probably not included in the present analysis.

In contrast with the exclusive effects of ST8SIA4 genetic depletion on dendritic complexity discussed above, both knockout strains show a common phenotype: a reduction of perisomatic inhibitory puncta on prefrontocortical pyramidal neurons. Our study suggest that this reduction affects mainly PV immunoreactive puncta, which most likely belong to fast-spiking basket cells. The decrease in the density of puncta immunoreactive for PV and SYN indicates that this is a reduction in active synapses. However, there is also a strong tendency towards a decrease in the density of PV+SYN− puncta, especially in *St8Sia2*^−/−^ mice. It should be noted that, unlike *St8Sia4*^−/−^, adult *St8Sia2*^−/−^ mice still express polySia in cortical interneurons (Nacher et al., 2010). In PV+ basket interneurons polySia can be found in some of the perisomatic puncta that these cells form around pyramidal neurons (Castillo-Gómez et al., 2008). Previous research in our lab showed that in the rat PFC around 70% of polySia+ puncta lacked SYN expression suggesting that these might be silent synapses. In any case, the decreases in perisomatic puncta observed in the present study contrast with the increased densities observed in response to an acute enzymatic depletion of polySia in the PFC *in vivo* during adulthood (Castillo-Gómez et al., 2011) or in organotypic cultures derived from early postnatal brain (Castillo-Gómez et al., 2016). It also contrasts with the inverse correlation between polySia expression and the establishment of inhibitory perisomatic input during maturation of the visual cortex (Di Cristo et al., 2007). Together, this points towards a differential role for the expression of polySia during early interneuron development as compared to postnatal maturation and adulthood, when the presence of this molecule appears to play an insulating role, restricting the connectivity of the polySia positive interneuron population (Castillo-Gómez et al., 2011; Nacher et al., 2013). Hence, the deficits in embryonic interneuron migration and the resulting decrease of interneuron densities in the mPFC (Kröcher et al., 2014) could be the underlying cause why *St8sia2*^−/−^ and *St8sia4*^−/−^ mice form less PV+ synaptic boutons on prefrontocortical pyramidal neurons.

In conclusion, the present study reveals an important impact of polySTs on the structure and connectivity of cortical interneurons. This is particularly relevant considering that genetic variation in both NCAM and these enzymes (especially *ST8SIA2*) has been associated with schizophrenia (Sullivan et al., 2007; Tao et al., 2007; Gilabert-Juan et al., 2013c), that *St8Sia2* knockout mice show schizophrenia-like phenotypes (Kröcher et al., 2015; Bacq et al., 2018), and that reductions in polySia-NCAM expression as well as alterations in inhibitory neurotransmission were detected in the PFC and hippocampus of schizophrenic patients (Barbeau et al., 1995; Gilabert-Juan et al., 2012) and in animal models of this disorder (Gilabert-Juan et al., 2013a; Castillo-Gómez et al., 2017).

## Data Availability

All datasets generated for this study are included in the manuscript.

## Author Contributions

JN and HH conceived the project. YC, JA and IR performed the experiments and supervised the breeding of the animals. JN supervised the project. YC, HH and JN wrote the article.

## Conflict of Interest Statement

The authors declare that the research was conducted in the absence of any commercial or financial relationships that could be construed as a potential conflict of interest.
